# How far can I reach? The perception of upper body action capabilities in Parkinson’s disease

**DOI:** 10.3758/s13414-021-02340-y

**Published:** 2021-07-06

**Authors:** Megan Rose Readman, Neil M. McLatchie, Ellen Poliakoff, Trevor J. Crawford, Sally A. Linkenauger

**Affiliations:** 1 Department of Psychology, Fylde College, Lancaster University, Bailrigg, Lancaster LA1 4YF UK; 2 Division of Neuroscience and Experimental Psychology, University of Manchester, Manchester, UK

**Keywords:** Parkinson’s disease, Movement disorder, Affordance perception, Perceptual-motor integration

## Abstract

Successful interaction within the environment is contingent upon one’s ability to accurately perceive the extent over which they can successfully perform actions, known as action boundaries. Healthy young adults are accurate in estimating their action boundaries and can flexibly update them to accommodate stable changes in their action capabilities. However, there are conditions in which motor abilities are subject to variability over time such as in Parkinson’s disease (PD). PD impairs the ability to perform actions and can lead to variability in perceptual-motor experience, but the effect on the perceptions of their action boundaries remains unknown. This study investigated the influence of altered perceptual-motor experience during PD, on the perceptions of action boundaries for reaching, grasping, and aperture passing. Thirty participants with mild-to-moderate idiopathic PD and 26 healthy older adults provided estimates of their reaching, grasping, and aperture-passing ability. Participants’ estimates were compared with their actual capabilities. There was no evidence that individuals with PD’s perceptions were less accurate than those of healthy controls. Furthermore, there was some evidence for more conservative estimates than seen in young healthy adults in reaching (both groups) and aperture passing (PD group). This suggests that the ability to judge action capabilities is preserved in mild to moderate PD.

**Supplementary Information:**

The online version contains supplementary material available at 10.3758/s13414-021-02340-y.

According to the ecological approach to visual perception (Gibson, 1979), successful interaction within the environment is contingent upon one’s ability to detect and select the affordances available within such an environment (Gibson, 1979). Affordances signify the reciprocal relationship between a given organism and its environment. That is, affordances are the opportunities for action for a given organism within a particular environment (Gibson, 1979; Heras-Escribano & Pinedo-García, 2018). Whilst an infinite number of affordances are present for any organism within an environment at any one time, the extent to which an object affords a specific behaviour is determined by the relationship between the specifications of the object and the morphological limitation of the perceiver’s body (Proffitt & Linkenauger, 2013). For example, the morphology of the human hand enables the performance of a grasping motion, yet constrains the range of object sizes over which this action can be performed. Therefore, as a consequence of morphology, one environmental feature can afford two entirely different behaviours to two different individuals.

The limits at which the successful performance of an action can no longer occur are known as action boundaries (Fajen, 2005). Successful interaction within the environment relies upon an individual’s ability to perceive such action boundaries accurately. Intuitively, this knowledge is acquired throughout childhood (Proffitt & Linkenauger, 2013). Specifically, 5-month-old infants perform hundreds of exploratory hand movements every 10 minutes (Wallace & Whishaw, 2003), transverse vast distances, and fall approximately 15 times per hour (Adolph et al., 2012). These exploratory movements provide infants with extensive visual feedback regarding what actions are possible and impossible. Which, in turn, facilitates the development of precise, fine-tuned knowledge regarding ones’ action boundaries (Proffitt & Linkenauger, 2013). Following development, adults are reliably in tune with their action boundaries, such that individuals are highly accurate at estimating the maximum step height that affords stepping (Warren, 1984), the farthest distance they can reach (Carello et al., 1989), the largest object they can grasp (Linkenauger et al., 2009), the smallest door opening they can pass through (Warren & Whang, 1987), and the smallest size opening they can fit their hand through (Ishak et al., 2014).

Whilst this research points towards individuals being reliably in tune with their maximal action boundaries present in stable environments, our bodies and the world in which we inhabit are continually changing, resulting in variations in one’s action boundaries (Franchak & Adolph, 2014a). Consider the rehabilitation period following an injury to the elbow that precludes arm extension. Immediately following the injury, the individuals’ ability to perform a reaching action will be severely compromised. However, during rehabilitation, the individuals’ ability to perform a reaching action will slowly recover in accordance with the healing of the injury. Therefore, in order for successful interaction within the environment to occur, it is imperative for individuals to detect varying constraints and update their action boundaries to account for such constraints.

Indeed, research has shown that healthy individuals can flexibly update their action boundaries to account for varying constraints (Proffitt & Linkenauger, 2013). For example, when hand size is enlarged by a prosthesis, the minimum size aperture participants attempt to fit their hand through increases in accordance with the increase in hand size (Ishak et al., 2008). Similarly, when the size of the hand is increased by magnification, participants subsequently perceived graspable objects to be smaller in size than when the hand was not magnified (Linkenauger et al., 2011). Additionally, the minimum doorway aperture perceived as passable increases in accordance with the increase in girth that occurs when individuals don a pregnancy pack (Franchak & Adolph, 2014b).

This evidence corroborates the notion that the perceptual system is in tune with one’s action boundaries and can flexibly update to accommodate for variance. But, this literature focuses on stable changes that allow individuals to gain relevant information regarding the visual specification of the altered action boundary. There are circumstances in which individuals action boundaries are not only permanently altered, but are also subject to fluctuations that are rapid and unpredictable in nature, thereby preventing learning of the visual specification of one’s altered action from occurring. A clear example of this occurs in people with Parkinson’s disease.

Parkinson’s disease (PD) is characterized by motoric atypicalities including tremor, rigidity (Berardelli et al., 1983; Politis et al., 2010), bradykinesia, hypokinesia, akinesia, and postural instability (Guttman et al., 2003). These motoric atypicalities characteristically impair the performance of many actions. For example, tremor of the hand is likely to constrict the individuals’ ability to grasp objects. Similarly, rigidity, both in the form of “lead pipe” rigidity, where a continuous resistance to movement throughout the range of motion is present (Guttman et al., 2003), and “cogwheel” rigidity, where patients’ ability to perform an action fluidly is replaced by small jerky movements (Ghiglione et al., 2005; Guttman et al., 2003), will restrict the individuals’ ability to perform various actions and reduce the range over which these actions can be performed. Importantly, when hypokinesia, the dismissed magnitude of the performance of movements (Berardelli et al., 2001; Simões & Litvan, 2010), occurs the patient’s muscular strength is preserved, and although access to motor programs can be delayed, access is still possible (Simões & Litvan, 2010). Therefore, whilst it is physiologically possible for the individual to perform an action over a certain range, in practice, execution of the action over this range cannot occur. These physiological reductions in the ability to perform actions and the range over which such actions can be performed will be accompanied by a reduction in the action boundary associated with the affected actions.

In addition to the reduction in the ability to perform actions, individuals with PD may receive inconsistent perceptual motor experience regarding what actions are possible and impossible. Characteristically, prior to diagnosis and during the earliest stages of PD, patients may experience unilateral symptom presentation; for example, whilst the left side of the body may be affected, the right side of the body may remain unaffected (Sveinbjornsdottir, 2016). When this arises, the individual will receive inconsistent perceptual-motor experience regarding the extent to which and the range over which they can perform actions based on which the side of the body they are using. Consider, for example, a patient with left-side lateralized rigidity performing a reaching action. The patient’s ability to perform a reach with the left arm will be severely compromised, whilst they will be able to perform a reaching action with the right arm to the maximum extent their morphology permits.

Dopaminergic medications, particularly Levodopa, are currently the “gold standard” treatment for PD (Dorszewska et al., 2014; Fahn, 2006). Levodopa treats the symptoms of PD by effectively replacing the loss of dopamine (Gandhi & Saadabadi, 2019) that occurs due to the degeneration of dopaminergic nigrostriatal neurons originating in the substantia nigra pas compacta of the basal ganglia and projecting to the striatum of the basal ganglia (Agid et al., 1987). Initially, dopaminergic medications offer substantial reductions in symptom intensity with very few adverse effects (Marsden & Parkes, 1977). However, following several years of levodopa therapy (Marsden & Parkes, 1977), according to Dupont et al. (1996), at least 50% of patients experience fluctuations in response to their dopaminergic medication throughout the course of a day. These fluctuations, in turn, may also produce fluctuations in the intensity of the motor symptoms displayed at different times even within a single day; this phenomenon is known as the on–off phenomenon (Bhidayasiri & Tarsy, 2012).

Notably, patients report that when they are in an “on” phase they can perform actions as normal; however, during “off” phase, their ability to perform motor actions is severely compromised (Lees, 1989). Some “off” periods may be predictable and related to the time of medication administration. For example, a patient may always have an “off” time at 3 p.m. (Stacy et al., 2005). Alternatively, some “off” periods may be highly unpredictable in both onset and duration (Lang et al., 1982). This means that individuals with PD will gain inconsistent perceptual-motor experience relating to their ability to perform an array of actions. Taken together this unstable variance, resulting from on–off symptom fluctuation and unilateral symptom presentation may affect a person with PD’s ability to accurately perceive their action boundaries for a range of actions.

Another reason that the perception of action capabilities may be affected in PD is due to changes in sensory and perceptual functions that occur as a consequence of changes in the basal ganglia in PD. Although the functional role of the basal ganglia has primarily been hypothesized to be motor (Schwarz et al., 1984), additional research highlights that the basal ganglia exert much wider functions in sensory and cognitive domains as well as motor (Haber & Gdowski, 2005; Marsden, 1982). For example, substantial deficits in basic visual processes such as light/dark adaptation, visual acuity, peripheral vision, and visual processing speed, have been observed in individuals with nontremor PD (Seichepine et al., 2011). Furthermore, deficits in visuospatial functions including distance perception (Davidsdottir et al., 2005), size perception (Lee et al., 2001), spatial navigation (Davidsdottir et al., 2008), spatial working memory (Kemps et al., 2005; Possin et al., 2008; Siegert et al., 2008), and spatial planning (Altgassen et al., 2007), have largely been observed in individuals with PD (Boller et al., 1984; Seichepine, 2012). Furthermore, Schneider, Diamond, and Markham (1986) showed that PD patients made significantly more errors in somatosensory tasks compared with age-matched healthy controls. As the perception of one’s action boundaries relies primarily on the integration of these sources of information, deficits in these processes could also lead to deficits in the ability to anticipate the range over which one can perform an action in PD.

Additionally, recent research points towards the notion that individuals with PD are not reliably in tune with the severity of the symptoms they present. That is, when both PD patients and clinicians are asked to rate the severity of the symptoms an individual is presenting, 30%–50% of nondemented, nondepressed PD patients indicate their symptoms to be less severe than clinicians’ ratings of them (Maier et al., 2012). Due to this partial lack of subjective awareness of motor deficits (Maier et al., 2012), it could be that some patients are not reliably in tune with their action capabilities as they fail to perceive the motor deficits they present.

The influence of natural variability on the subsequent perception of one’s action boundaries has yet to be investigated. However, we can draw on insights obtained from analyses of the effect of artificial variability to inform how we may anticipate individuals’ perceptions of their action capabilities to be influenced by natural variability that may occur in PD. For example, Lin et al. (2020) observed that when participants’ reaching ability varied from 50% to 150%, from reach to reach, individuals displayed a bias towards liberal estimations of their action boundary. Notably, this effect was observed regardless of whether the variability was completely random or systematic. Furthermore, Readman et al. (2021) observed that when grasping ability varied from 50% to 100% to 150% from grasp to grasp, so that participants gained equal experience with all grasping capabilities, participants estimated their action boundary to be similar to the normal condition. Similarly, when variability was systematic, so that participants gained more perceptual-motor experience with the extended grasp (150%), participants also estimated their action boundary to be the normal grasp.

Based upon these findings we may anticipate that individuals with PD’s perceptions of their action boundary for reaching would be more liberal, and thereby less accurate, than typically ageing individuals. However, regarding the perception of one’s grasping ability, we may anticipate that PD patients will calibrate to the middle of all experience they have gained—that is, both during “on” and “off” times, which presumably would be their true morphologically derived action boundary. Consequently, PD patients’ subsequent perceptions of their action boundary for grasping may not significantly differ from healthy age-matched controls who do not experience this variability.

The incongruence of the results obtained concerning the influence of artificial variability may be taken to indicate that the perceptual system does not inevitably employ the same mechanism in the face of variability irrespective of the action in question. Therefore, we may anticipate that natural variability in one’s perceptual-motor experience as a consequence of PD, may differentially influence PD patients’ perceptions of their action boundaries based on the action in question. Therefore, in addition to the primary research aim, this study will also address a further question: Is the effect of PD on the perception of one’s action boundaries the same regardless of the action in question?

To address these questions individuals with mild-to-moderate idiopathic PD and healthy ageing controls estimated the maximum extent to which they can perform reaching, grasping, and aperture-passing actions. Participants’ estimations of their action capabilities were then compared with their actual ability.

## Method

### Participants

G*Power software (Faul et al., 2007) was used to perform an a priori power analysis to ascertain the required sample size in order to achieve adequate power. Three individual power analyses, for each of the three tasks employed, were performed. The required power (1 − β) was set at .80 and the significance level (α) was set to .05. The individual effect sizes for each task were based on Graydon et al. (2012), who employed the same methodology as employed here. For the reaching ability task, we anticipated a medium effect size of 0.37. Therefore, for the frequentist parameters defined, a sample size of *N* = 8 (four per condition) is required to achieve a power of .80 at an alpha of .05. For the grasping ability task, we anticipated a large effect size of 0.60. Therefore, for the frequentist parameters defined, a total sample size of *N* = 70, *N* = 35 per condition, is required to achieve a power of .80 at an alpha of .05. For the aperture passing task, we anticipated a small effect size of 0.18. Therefore, for the frequentist parameters defined, a total sample size of *N* = 36 (18 per condition) is required to achieve a power of .80 at an alpha of .05.

Unfortunately, due to the COVID-19 global pandemic, the sample size recruited, and subsequent data analyzed, was smaller than necessary in order to achieve adequate power for the grasping task (*N*_PD_ = 19, *N*_Healthy older adult controls_ = 21; *but* only the grasping task). However, the sample size recruited was greater than that of the previously validated Graydon et al. (2012) study.

Thirty patients with idiopathic PD (10 females), and 26 healthy older adult controls (15 females) participated. The mean age between the two groups did not significantly differ, *t*(54) = −1.198, *p* = .236. Fifty-one (27 PD patients) participants were right-handed, four (two PD patients) were left-handed, and one PD patient was mixed-handed (Oldfield, 1971). The one mixed-handed participant elected to complete the task with their left hand. All participants had normal or corrected-to-normal vision with a visual acuity between 20/20– 20/30 in both the left and the right eye, as classified by the Snellen chart.

Participants were screened for the presence of cognitive impairment using the Montreal Cognitive Assessment (MOCA; Nasreddine et al., 2005). The MOCA was used because previous research has shown that it is perhaps the most sensitive cognitive examination for screening for mild cognitive impairment in the presence of PD (Dalrymple-Alford et al., 2010; Hoops et al., 2009; Kandiah et al., 2014). Participants’ data were included in analysis only if they scored within the normal range (≥26 out of 30). Following this exclusion criterion 13 (10 PD patients) participants’ data were removed prior to analysis. Average MOCA scores did not significantly differ between patients and controls, *t*(41) = −.836, *p* = .408. One control participant indicated a history of a neurological illness; therefore, their data were removed prior to analysis. Subsequently following exclusion on these grounds, 42 (20 PD) participants’ data were included in the following analyses.

Of the 42 participants whose data were included in analysis, 11 participants (five PD patients) indicated they had a current or history of a diagnosis of rheumatic illnesses, 10 participants (four PD patients) disclosed that they had a history of a diagnosis of a psychiatric illness, including depression (three PD patients, four controls), and anxiety (one PD patient, two controls). All participants were screened for the presence of depression and anxiety using the Hospital Anxiety and Depression Score (HADS; Zigmond & Snaith, 1983; see Table 1, for HADS data).

**Table 1:** The mean (*SD*) background characteristics for the Parkinson’s disease (PD) and control groups

Group	PD	Control
Age	65.85 (7.21) Range: 54–76	67.86 (6.84) Range: 54–77
MOCA	27.60 (1.27) Range: 26–30	27.91 (1.51) Range: 26–30
HADS–Anxiety	6.50 (4.523) Range: 0 -15	6.27 (4.05) Range: 1–15
HADS–Depression	4.13 (2.50) Range: 1–9	1.77 (1.60) Range: 0–6
Years since diagnosis	4.26 (4.41) Range: 0.833–17	
MDS-UPDRS Motor examination	36.20 (7.81) Range: 24–50	
MDS-UPDRS Motor complications	3.20 (3.12) Range: 0–9	
Hoehn and Yahr stage	1.65 (.75) Range: 1–3	
Years on medication	4.09 (4.13) Range: 0.833–15	
Time since last dosage of medication (minutes)	146.94 ( 83.96) Range: 0–300	
L-Dopa dosage (mg)	477.88 (255.04) Range: 0–1290	

PD patients were selected who were at a Hoehn and Yahr Stage 3 or less. The Hoehn and Yahr stage provides an overall summary of the severity and laterality of symptoms presented by the individual with Parkinson’s. Ten patients presented unilateral symptoms only (Stage 1), seven patients presented symptoms bilaterally but with no impairment of balance (Stage 2), and three patients displayed bilateral symptoms with some postural instability but were physically independent (Stage 3). Parkinsonian symptoms were assessed using the motor examination and the motor complication subscales of the Movement Disorder Society–Unified Parkinson’s Disease Rating Scale (MDS-UPDRS; Goetz et al., 2008). All but one PD patients were receiving parkinsonian medication and were tested under their usual medication regime. Twelve patients indicated that they experienced motor fluctuations. All of these patients were in a typical functioning “ON” phase at the time of testing. Eighteen patients were taking combination drugs (containing levodopa and a peripheral dopa-decarboxylase inhibitor; e.g., Madopar), five patients were taking a dopamine agonist (e.g., Ropinirole), five patients were taking a monoamine oxidase inhibitor (e.g., Rasagiline) and one patient was taking a Catechol-O-Methyl Transferase (e.g., Entacapone; see Table 1 for patient characteristics).

PD patients were recruited through the Royal Preston Hospital, and through advertisement with Parkinson’s UK. The healthy controls were either the partners or relatives of the PD patients or were recruited through the ageing research database at Lancaster University. Testing occurred at either the clinical research facility at Royal Preston Hospital or in the adult testing facilities at Lancaster University. This study was approved by the local National Health Service (NHS) ethics committee.

### Stimuli and apparatus

Participants completed all three tasks sat at a chair positioned an arm’s length away from a standardized table (140 cm × 80 cm).

#### Task 1: Perception of reaching ability

**Fig. 1:**
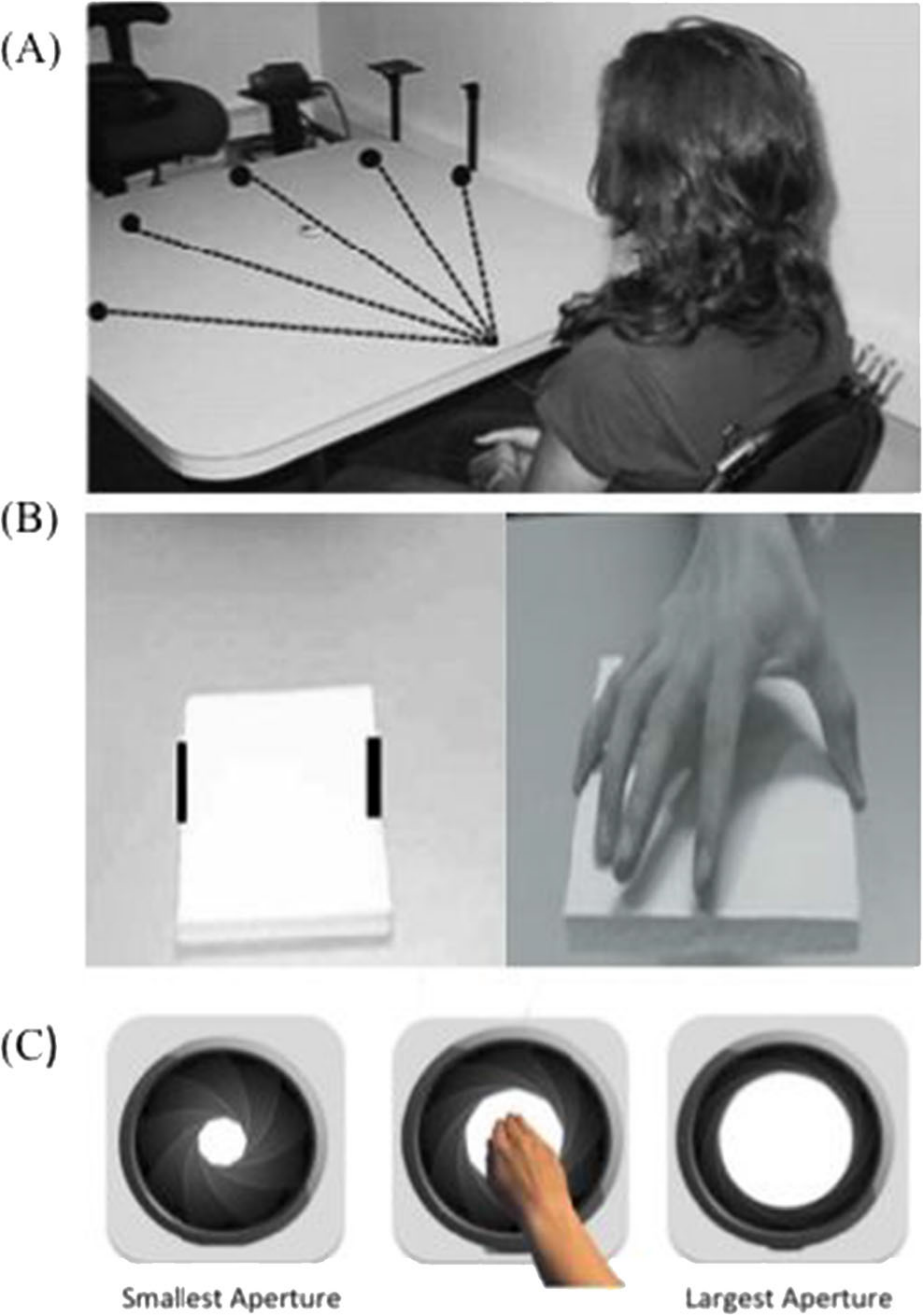
Visual illustrations of the (**a**) reaching ability task, (**b**) grasping ability task, (**c**) aperture passing task. **a** The solid dots represent the 30°, 15° unilateral/ipsilateral and centre axis stickers. The dotted black line represents the axis along which the chip was moved either towards or away from the participant. **b** The black lines on the parallel edges of the block were where the participant was asked to imagine extending their hand from and to when estimating grasping ability and were where participants were told to place their fingers when deducing actual grasping ability

Five axis stickers placed at 30^o^ and 15^o^ to the left, at the centre, and 15^o^ and 30^o^ to the right, were placed on the far side of the table. A sixth origin sticker was located directly in front of the participants’ torso (see Fig. 1). Reaching judgements were made using a green chip that was moved towards and away from the participant along a diagonal specified by an axis sticker and the origin sticker.

#### Task 2: Perception of grasping ability

A set of 16 1cm thick foam board square blocks, were used as the graspable stimuli. The width of these square blocks ranged from 4- 25cm and increased in 1.4cm increments. Each block had two parallel black lines (3cm) long drawn in the centre of opposing sides, this occurred to indicate where the participant was to imagine placing their finger and thumb when grasping the object (See Figure 1).

#### Task 3: Perception of aperture passing

A portable apparatus with an easily manipulated aperture was created. This apparatus was made up of 3D printed black triangles that open and close alike to a camera lenses aperture. All 3D printed components were attached to a grey wooden frame. The size of the aperture was manipulated by moving a handle towards the right to create a larger aperture and towards the left to create a smaller aperture (see Fig. 1).

### Procedure

To commence the testing session participants were screened for mild cognitive impairment and depression and/or anxiety, and background cognitive and health measures were obtained. PD patients’ Parkinsonian symptoms were assessed using the motor examination and motor complications subscales of the MDS-UPDRS. The order in which the participants completed the three tasks was counterbalanced.

#### Task 1: Perception of reaching ability

Participants sat an arm’s length away from the table, with the back of their clothing clipped to the chair so that their shoulders were held against the back of the chair, and their hands on their lap (see Fig. 1). This occurred to serve as a constant reminder of the range of motion that they were to use when making their estimates of anticipated reach and to ensure that all participants estimated reachability in the same way. Participants were informed that they would be required to estimate their maximum reaching ability for all diagonals. At no point before providing their estimations were participants allowed to overtly perform a reaching movement over the table. This precaution prevented participants from receiving confirmatory information about their actual abilities prior to their estimates. The researcher then moved a 1-inch green chip either towards or away from the participant, along one of the diagonals specified by an axis sticker and the origin (see Fig. 1a). Participants were asked to indicate when the chip was just in reach of their dominant hand, whilst maintaining the specified posture. Participants were encouraged to ask the researcher to adjust to the chip’s location to ensure the estimate of reaching ability was as accurate as possible.

To control for hysteresis, the starting position of the green chip was either directly in front of the participant, at the origin sticker, or at the end of the movement axes, and moved both towards and away from the participant for each of the five diagonals. Therefore, participants made 10 reachability estimations. The order of trials was counterbalanced across participants. When the chip was moved away from the participant, the chip started at the origin sticker and was moved towards one of the axis stickers. When the chip was moved towards the participant, the chip started at one of the axis stickers and was moved towards the origin sticker.

Once participants were satisfied that the chip was located in the correct position, participants were instructed to close their eyes, and the distance from the origin to the centre of the chip was measured and recorded. Participants were required to close their eyes in order to prevent feedback regarding the distance of reachability being obtained and used in later trials.

On completion of all perceived reachability trials, a measure of actual reachability for each diagonal was obtained. To do so, participants were instructed to move the chip as far away as they could along one diagonal whilst maintaining the specified posture.

#### Task 2: Perception of grasping ability

Participants were seated at the standard table and instructed that they would be required to estimate whether they could grasp a series of blocks with their dominant hand. Grasping was defined as the ability to place their thumb on the black line on one edge of the block and extend their hand over the surface of the block so that one of their fingers was placed on the black line on the parallel edge of the block. Participants were asked to close their eyes whilst the researcher placed one of the 16 blocks on the table perpendicular to the participant. Participants were asked to close their eyes at this time in order to prevent them from gaining visual information regarding the researcher’s ability to grasp the blocks and subsequently use this information to guide their grasping-ability estimations. Once the block had been placed, participants were instructed to open their eyes and use visual inspection only to indicate whether they would be able to grasp the block with their dominant hand. This procedure occurred for all 16 blocks, and the order of completion was counterbalanced across participants. On completion of all estimation trials, a measure of actual grasping ability was obtained. This was obtained by asking participants to overtly grasp the largest block they could with their dominant hand. Participants were encouraged to try the next size up to ensure that a true measure of maximal grasping ability was obtained.

#### Task 3: Perception of aperture passing

Participants were seated at a standard table, upon which the aperture passing apparatus was located in the centre of the table (see Fig. 1c). Participants were instructed to estimate the point at which they could just fit their dominant hand through the aperture without coming into contact with the black inner triangles, whilst keeping their hands on their lap. Participants were asked to imagine performing the aperture-passing movement with their hand with their fingers closed. Participants completed four trials; in two trials, participants were presented with the largest size aperture, and the researcher gradually made the aperture smaller. In the remaining two trials, the participant was presented with the smallest aperture, and the researcher gradually increased the aperture size. At the point at which the participant indicated to the researcher they could just fit their hand through the hole, the participant was instructed to close their eyes and the researcher measured the aperture. Participants were instructed to close their eyes to prevent them from gaining visual feedback on the aperture size and using this information in later trials. Following the perceived aperture-passing trials, a measure of smallest aperture size that the participant could actually fit their hand through was obtained. To obtain this, participants were asked to place their hand in the hole, and the researcher gradually reduced the size of the aperture to the point at which the hand just fitted in the aperture without coming into contact with the black triangles.

### Data analysis

For each of the three tasks, we report independent-samples *t*-test analyses of differences in the actual abilities of PD patients compared with healthy older adult controls. The accuracy of the perceived action boundary was measured by calculating the ratio of the estimated ability, to the actual ability. A value of more than 1 indicates that the participant overestimated their ability, whilst a value of less than 1 indicates the participant underestimated their ability. For reaching ability, this ratio was calculated for each of the five diagonals independently. The accuracy ratios were then compared between the PD and healthy controls (reaching: mixed ANOVA; grasping: independent-samples *t*-test; aperture passing: mixed ANOVA).

As a single nonsignificant *p*-value cannot be used to infer evidence for the null hypothesis (for a further discussion, see Lakens et al., 2020), we also report Bayes factors for all 1-*df* analyses. Bayes factors provide a continuous measure of evidence regarding how well the data were predicted by one hypothesis (e.g., the null; H0), relative to another hypothesis (e.g., the alternative; H1). We calculate Bayes factors using the Dienes and McLatchie (2018) R script calculator and follow Jarosz and Wiley’s (2014) thresholds, and interpret Bayes factors between 0.33 and 3 as weak and inconclusive, Bayes factors between 0.05 and 0.33 and 3 and 20 as moderate evidence for the null and experimental hypotheses, respectively, and Bayes factors <0.05 and >20 as strong evidence for the null and experimental hypotheses, respectively. Bayes factors require one to specify an approximate scale-of-effect predicted by one’s theory, and we specify in the footnotes throughout each Results section the prior research we use to specify our scale-of-effect. Lastly, we report robustness regions to indicate the sensitivity of the categorical conclusions drawn from the Bayes factors to the approximate scale-of-effect used. Robustness regions are reported as *RR*(*S*, *L*), where *S* corresponds to the smallest scale-of-effect and *L* to the largest scale-of-effect that would still yield the same conclusion.

Previous studies have shown that anxiety significantly influences participants’ perceptions of their action boundaries for reaching behaviours (Graydon et al., 2012). Given that anxiety disturbances are recognized as one of the most common nonmotor comorbidities of PD (Chen & Marsh, 2014), a mixed analysis of covariance (ANCOVA) was completed in order to ascertain the potential influence of anxiety, as measured by the HADS, on participants perceptions of their action boundaries. Across all three tasks, anxiety did not significantly influence individuals’ perceptions of their action capabilities (see Appendix 1 for the full statistical analysis).

Furthermore, arthritis can affect both overt movement and motor imagery (the ability to mentally rehearse actions; Gandola et al., 2017; Sacheli et al., 2018). Therefore, a mixed ANCOVA was completed to ascertain the potential influence of the presence of rheumatic illnesses on participants’ perceptions of their action boundaries. Across all three tasks, the presence of arthritis did not significantly influence individuals’ perceptions of their action capabilities (see Appendix 2 for full statistical analysis). Therefore, both anxiety and the presence of rheumatic illnesses should not be considered confounding factors in this analysis.

### Results

#### Task 1: Perception of reaching ability

Forty participants (18 PD patients) were included in this analysis. One PD patient’s data were removed for providing estimations that were ±2 standard deviations away from the mean, and one PD patient failed to fully complete the reaching ability task

There was no significant difference between the average actual reaching ability (across the five diagonals) of PD patients (*M =* 46.16*, SD =* 5.49*)* compared with the reaching abilities of healthy older adult controls (*M =* 43.48*, SD=* 5.37), although the evidence for the null hypothesis was only weak, *t*(38) = 1.55, *p* = .129, *B*_*N(0,8.35)*_^1^ = 0.64, *RR*[0, 16.59].

**Fig. 2:**
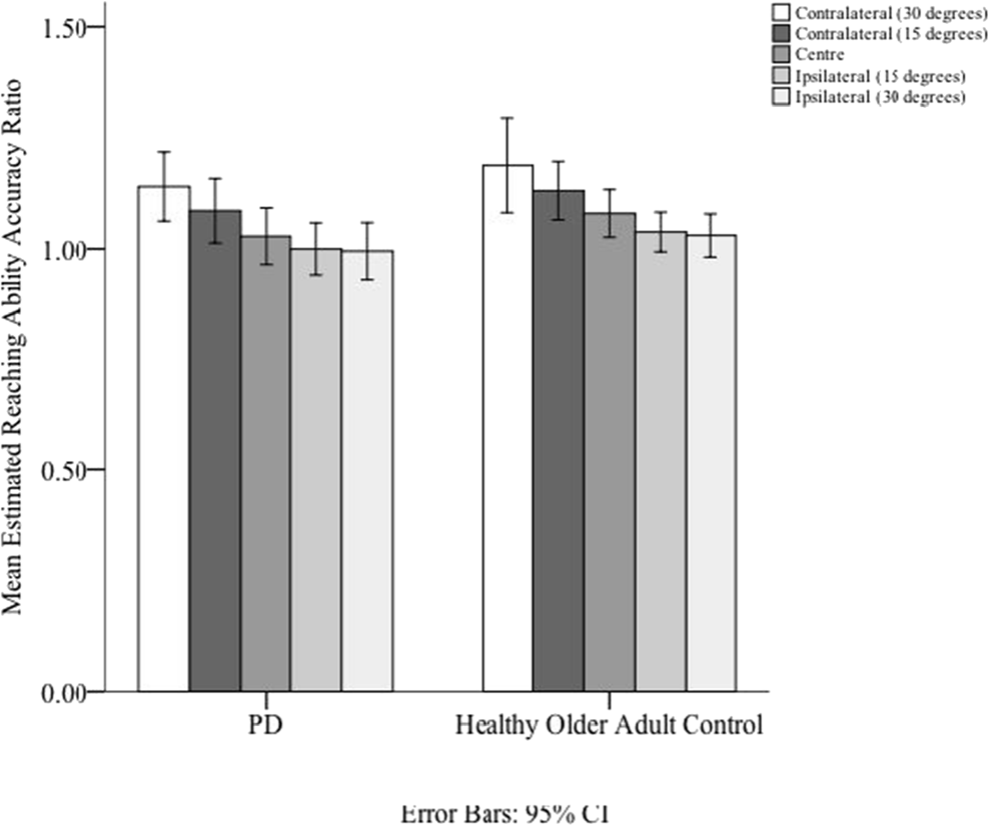
Means (and standard deviations) of estimated/actual reaching ability ratios for each diagonal. Error bars represent 95% confidence intervals, calculated within subjects for each condition

The perception of the action boundary for reaching was analyzed by a mixed ANOVA [Diagonal direction (30^o^ contralateral, 15^o^ contralateral, directly in front, 15^o^ ipsilateral, 30^o^ ipsilateral) × Group (PD or typically ageing older adult)]. A Greenhouse–-Geisser correction was applied to correct for violations of sphericity. A significant main effect of diagonal direction on perceived action boundary for reaching was observed, *F*(2.039, 77.497) = 19.087, *p* < .001, η_p_^2^=. 33). Participants overestimated contralateral estimates (*M*_D1_ *=* 1.16, *SE*_D1_ *=* .033; *M*_D2_ *=* 1.11, *SE*_D2_ *=* .023) more than ipsilateral estimates (*M*_D4_ *=* 1.02, *SE*_D4_ *=* .017; *M*_D5_ *=* 1.01, *SE*_D5_ *=* .019; see Fig. 2).

**Fig. 3:**
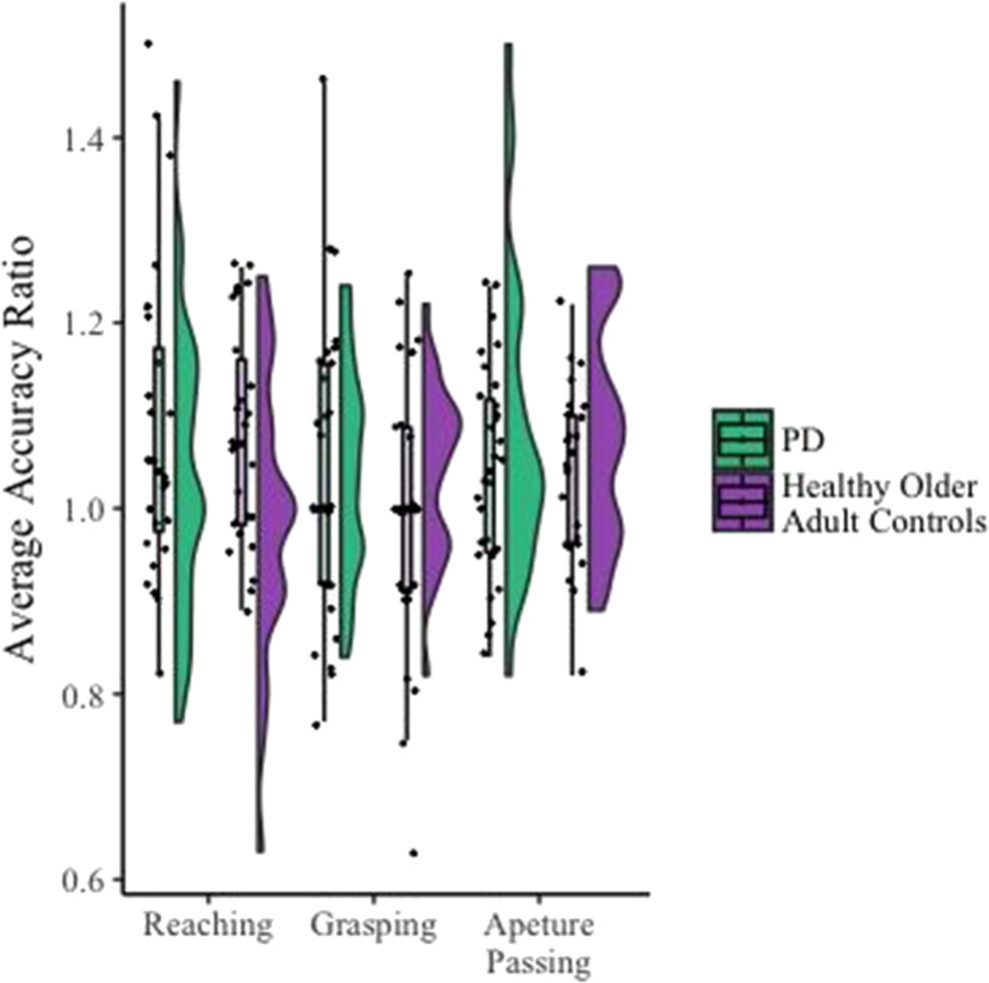
Group means (and standard deviations), data distribution, and jittered raw data (raincloud; each dot represents an individual participant) of estimated/actual reaching, grasping, and aperture-passing ability ratios for the PD and healthy older adult control groups. Error bars represent ±2 *SEM*, calculated within each condition. There was no significant difference in accuracy ratio between people with Parkinson’s and those without (reaching ability; *p* = .260, grasping ability *p* = .882; aperture passing ability *p* = .760; see text for details)

There was no significant difference in the accuracy of the perceived action boundaries for reaching between the PD (*M*_acc_= 1.050, *SE*_acc_ = .028) and healthy older adult groups (*M*_acc_= 1.093, *SE*_acc_ = .025), *F*(1, 38) = 1.309, *p* = .260 (see Fig. 3), although the Bayes factor indicated that the evidence only weakly favoured the null, *B*_*H(0,0.09)*_ = 0.70, *RR*[0, 0.21].^2^

#### Task 2: Perception of grasping ability

Forty participants’ (19 PD patients) data were included in the final analysis. Two (one PD patient) participants’ data were removed prior to analysis because they provided estimations that were ±2 standard deviations away from the mean.

There was no significant difference and moderate evidence for the null when comparing the physical actual grasping ability of PD patients (*M* = 16.16, *SD* = 1.81 ) compared with the physical actual grasping ability of healthy older adult controls (*M =* 15.93, *SD=* 1.57), *t*(38) = .42, *p* = .677, *B*_*N(0,4*)_^3^ = 0.15, *RR*[1.69, ∞].

An independent-samples *t* test revealed no significant difference between the accuracy of the perceived action boundary for grasping between the PD (*M*_acc_ = 1.017, *SD*_acc_ = .114) and healthy older adult groups (*M*_acc_= 1.011, *SD*_acc_ = .125), *t*(38) = .76, *p* = .882 (see Fig. 3). Bayes factor indicated that the evidence provided moderate support for the null, *B*_*H(0,0.08)*_ *=* 0.13, *RR*[0.03, ∞].^4^

#### Task 3: Perception of aperture passing

All 42 participants’ (20 PD) data were included in the analysis. An independent-samples *t* test revealed that there was no significant difference and moderate evidence for the null hypothesis when comparing the actual aperture-passing ability of PD patients (*M =* 8.84, *SD =* .90*)* compared with the aperture-passing abilities of healthy older adult controls (*M =* 8.56, *SD=* .77), *t*(40) = 1.05, *p* = .298, *B*_*N(0,4.28)*_^5^*=* 0.11, *RR*[1.32, ∞].

A repeated-measures ANOVA [Initial aperture size (Small or Large) × Group (PD or typically ageing older adult)] indicated that there were no significant differences in the perceived action boundary for aperture passing between the PD patients (*M*_acc_ = 1.043, *SE*_acc_ = .022) and the healthy older adult controls (*M*_acc_ = 1.053, *SE*_acc_ = .021), *F*(1, 40) = .094, *p* =.760 (see Fig. 3). Bayes factor indicated that the data provided only weak evidence for the null hypothesis that patient accuracy for aperture did not differ from the control accuracy, *B*_*H(0,0.08)*_ *=* 0.39, *RR*[0, 0.09].

A significant main effect of hysteresis was observed, *F*(1, 40) = 33.377, *p* < . 001, whereby participants overestimated the minimum size opening they could successfully pass their hand through to be larger when the aperture started at the largest size and moved inwards (*M*_acc_ = 1.074, *SE*_acc_ = .017), than when the aperture started at the smallest size and moved outwards (*M*_acc_ = 1.022, *SE*_acc_ = .015).

#### Across all three tasks

Across all three tasks we found no significant difference in the accuracy of individuals with PD’s perceptions of their action boundaries compared with healthy older adult controls (see Fig. 3). Additionally, in Tasks 1 and 3, Bayes factors indicated that the evidence only weakly favoured the null hypothesis, whereas in Task 2 the Bayes factor indicated that the evidence moderately favoured the null hypothesis.

Visual analysis of the accuracy ratios obtained within these tasks compared with the accuracy ratios obtained in previous studies, recruiting young adult samples (such as Graydon et al., 2012), indicate that overall, both PD patients’ and healthy older adults’ perceptions of their action boundaries are more conservative than younger controls. Analysis of variance on the summary data (means and standard errors) obtained in this study compared with Graydon et al. (2012) show that healthy older adults (*M*_Control_ = 1.093 , *SE*_Control_ = .025) and individuals with PD (*M*_PD_ = 1.050, *SE*_PD_ = .028) overestimated their reaching ability significantly less often than did younger adults (*M =* 1.21, *SE* = 0.03; *p* = .014 and *p* < .001, respectively). Similarly, individuals with PD (*M*_PD_ = 1.043, *SE*_PD_ = .022) overestimated their aperture passing ability significantly less than younger adults (*M =* 1.14, *SE* = 0.04; *p* = .045). However, healthy older adults (*M*_Control_ = 1.053 , *SE*_Control_=.021) did not differ significantly from younger adults (*p* = .073) in their aperture passing ability . Furthermore, both healthy older adults (*M*_Control_ = 1.011 , *SE*_Control_ = .125) and individuals with PD (*M*_PD_ = 1.017, *SE*_PD_ = .114) did not differ from younger adults (*M* = 1.10, *SE =* 0.03; *p* = .838 and *p* = .863, respectively) in their estimation of their grasping ability.

Exploratory correlational analyses, with a Bonferroni correction for multiple comparisons, were conducted to analyze the influence of specific disease characteristics on individuals’ perceptions. No clinical disease related characteristics significantly correlated with perceived reaching ability, grasping ability and aperture passing ability accuracy (see Appendix 3), although the Bayes factor robustness regions indicated the correlational data were inconclusive for all models of H1 specified with scale-of-effects ranging from zero to large correlations (e.g., *r*s > .60). The only exception was that the correlation between years on medication and aperture accuracy estimate ratio provided strong evidence for the alternative hypothesis, *B*_*N(0,0.2)*_ = 95.07, *RR*[.09, .63].

## Discussion

The influence of altered perceptual-motor experience associated with PD on perceptions of their action boundaries was examined for upper body actions across three tasks. The findings obtained indicate that both PD patients and healthy older adult controls perceptions of their action capabilities for reaching are more conservative than healthy younger adult controls. Similarly, individuals with PD’s perceptions of their aperture-passing capabilities were more conservative than those of healthy younger adult controls. However, both individuals with PD and healthy older adult controls perceive their grasping capabilities comparably to healthy younger controls. Importantly, relating to our key interest, we observed that despite the reduced ability to perform actions and the natural variability in perceptual-motor experience relating to one’s ability to perform actions that may occur in PD, no significant differences from the control group in terms of the accuracy of one’s perceptions were observed. We will first consider why both PD patients and healthy older adult controls’ perceptions of their action capabilities are more conservative than younger adults before considering overall why individuals with PD’s ability to accurately perceive their action capabilities are preserved.

Consistent with the vast body of literature, which has shown that individuals overestimate their reaching (Fischer, 2000; Linkenauger et al., 2009), grasping (Linkenauger et al., 2009; Linkenauger et al., 2011), and aperture passing abilities (Graydon et al., 2012), both PD and healthy older adult controls overestimated their action boundaries for these actions. However, the magnitude of overestimation obtained here regarding reaching compared with previous studies, which typically recruit young adults, suggests that both people with PD and healthy older adults are more conservative in their estimations of their action boundaries for reaching than healthy younger controls. Similarly, individuals with PD, but not healthy older adult controls, are more conservative in their estimations of their action boundaries for aperture passing. Intuitively, it would be advantageous for older adults to be more conservative when estimating the maximum extent to which they can perform an action. Ageing is associated with a decline in muscular strength (Hunter et al., 2016), the speed at which motor actions are performed (Voelcker-Rehage, 2008), and the accuracy of motor control (Rodrigue et al., 2005). Consequently, older adults may be more risk averse than younger adults and tend towards more conservative estimations of their action boundaries.

However, importantly, the healthy older adult group were not more conservative in their estimations of their action boundaries for aperture passing and both individuals with PD and healthy older adults estimate their action boundaries for grasping in a comparable way to healthy young adult controls. This may in part be due to the nature of the action in question. Specifically, reaching and aperture passing are ballistic movements that act to support more intricate actions, such as grasping (Jeannerod, 1996). Due to these differential mechanical demands on the body, reaching, grasping, and aperture-passing behaviours will carry differential cost-benefit ratios (Franchak & Adolph, 2014a). Specifically, as reaching and aperture passing support more intricate actions such as grasping, if failure to perform a reach or aperture-passing movement occurs, the individual will also be prevented from performing the more intricate movement the reach or aperture passing movement supports. As a result, failure to perform reaching and aperture passing movements may be more consequential than grasping movements. Previous research has indicated that individuals’ perceptions of their action capabilities take into consideration the likelihood of success compared with the cost of failure (Franchak & Adolph, 2014a). Therefore, it may be that older adults and individuals with PD are more cautious in their estimations of their action capabilities for reaching and aperture passing but not grasping due to the costs associated with the failure of performance of these actions.

However, as this study did not directly analyze the influence of ageing on perceptions of action boundaries, these conclusions are somewhat speculative and should be approached with caution. Further research that recruits a sample spanning from younger adults (or perhaps children) to older adults and analyzes the influence of ageing on individuals’ perceptions of their action boundaries is required.

Due to lack of difference between the accuracy ratios for PD patients and healthy older adult controls across all three experiments, our findings indicate that people with mild-to-moderate PD perceive their action boundaries in a comparable way with healthy age-matched controls, despite their altered motor experience. Additionally, the correspondence of the results obtained across all three tasks can be taken to indicate that the effect of PD is the same across the three upper body tasks analyzed. However, Bayes factors for reaching ability and aperture-passing ability indicated that the evidence was only weakly in favour of the null hypothesis that PD does not influence perceptions of individual’s action boundary for reaching and aperture passing. Comparatively, regarding grasping, Bayes factors provided moderate support for the null hypothesis. Furthermore, correlational analyses revealed no significant correlations between specific disease characteristics and average estimated/actual ability accuracy ratio. Although it is worth noting that the current experiment was somewhat underpowered to detect anything but large correlations, and Bayes factors confirmed that all correlations were inconclusive. Furthermore, as no significant differences between the accuracy of PD and healthy older adults’ perceptions of their action capabilities when anxiety was controlled for as a covariate were observed, we can reasonably conclude that anxiety did not significantly influence the pattern of results.

It is important to note that the grasping task was slightly underpowered due to the sample size recruited being smaller (*N* = 40) than suggested by priori power analyses (*N* = 54). This is problematic because not only do analyses of the results obtained in underpowered studies often result in biased conclusions being drawn (Crutzen & Peters, 2017), the parameters computed from the limited samples may differ from the overall population (Crutzen & Peters, 2017). This could mean that it is not appropriate to draw conclusions based on the grasping task employed here. However, the Bayes factor on the results obtained in the grasping task provides moderate support for the null. Consequently, there is support for the conclusion that PD does not significantly influence perceptions of action boundaries for grasping.

Although some evidence shows that certain individuals with PD show impaired awareness of their motor symptoms (Maier et al., 2012), it is also possible that other PD patients are more consciously aware of, and pay more attention to, their action capabilities and thus may be more reliably in tune with their action boundaries. Consistent with this, Proffitt and Linkenauger (2013) argue that it is the exposure to the visual specification of actions that are possible and impossible that enables individuals to be reliably in tune with their action boundaries. Presumably, if individuals with PD are more consciously aware of, and pay more attention to their action capabilities, they will have enhanced exposure to the visual specifications of actions that are possible and impossible, causing them to be reliably in tune with their action boundaries. Corroborating this, Ramenzoni et al. (2010) observed that healthy young participant’s estimates of their action boundaries became more accurate over trials in which they were provided with optical information regarding their action boundary.

Previous research has also shown that individuals with PD simulate imagined movements (motor imagery; MI) comparably to their current motor capabilities (Abbruzzese, Avanzino, Marchese, & Pelosin, 2015). For example, Heremans et al. (2011) observed that whilst MI for individuals with PD is slower, MI was slowed to the same extent that physical execution was slowed (see also Avanzino et al., 2013; Dominey, et al., 1995). As MI is slowed to the same extent as physical motor performance is slowed, the slowness in MI appears reflective of the symptoms of PD rather than impairment in MI (Caligiore et al., 2017; Poliakoff, 2013). Furthermore, normal performance has been observed in tasks such as the hand rotation task, in which external stimuli implicitly demand the use of MI (Scarpina et al., 2019). In the current task, external objects provide a stimulus towards which an action can be imagined, and therefore motor imagery may be preserved.

Furthermore, MI in PD also reflects whether the individual is in the “on” or “off” phase. That is, if the participant was physically incapable of performing the action whilst in an “off” phase, they were also unable to imagine performing the action in this time (Dominey et al., 1995). Concerning the current study, all participants reported that they were currently in an “on” phase at the time of participation. Therefore, one would anticipate that their estimates would have been in keeping with their action boundary whilst in an “on” phase. Future research could explore whether their estimates change when tested off medication and/or directly compare limbs in people with asymmetrical PD.

Furthermore, whilst individuals can seemingly fluctuate from an “on” to an “off” time throughout the course of the day (Lang et al., 1982; Stacy et al., 2005), the stable maintenance of blood plasma levodopa concentration provided by medication reduces swings in motor performance (MacMahon et al., 1990), ensuring that patients spend more time in an “on” time throughout the course of a day. Within the sample tested here, 40% of patients reported they had no on/off time, 45% spent ≤25% of their waking hours in an “off” state, and the remaining 15% spent 26%–50% of their waking hours in an “off” state. Consequently, the individual will gain a greater array of visual information regarding their action capabilities when they are in an “on” time than when they are in an “off” time. As the majority of the learning required in order for one to be reliably in tune with their action boundaries occurs in an “on” phase, when individuals are asked to estimate their action boundaries, patients may disregard the limited amount of visual information obtained regarding their action boundaries in an “off” state in favour of the more fruitful information regarding their action boundaries in an “on” phase. If this is the case, then it would be logical for their estimations to reflect their abilities during an “on” phase. As individuals can typically perform all actions as normal to their maximal boundary when functioning in an “on” phase (Lees, 1989), their subsequent perceptions of their action boundaries should not differ from that of healthy older adults who do not have this source of variability present.

Alternatively, the perceptual system may apply a mechanism based on weighted averages when determining the action boundary for the action in question. According to this mechanism, the perceptual system will take into consideration all prior experience weighted by their occurrence and calibrate to the average (Körding & Wolpert, 2006). For example, if a patient can perform a grasp that is 100% of their ability 75% of the time, whilst the remaining 25% of the time they can only perform a grasp 50% of their maximal ability. When the patient is then asked to estimate their action boundary, they will calibrate to the average of all perceptual motor experience, 87.5% of their maximal ability, to inform their estimation. Regarding the sample tested within this series of studies, as the majority/all patients experience a greater proportion of “on” time than “off” time, the calculated weighted average for all participants will fall substantially closer to the participants maximal morphologically dictated action boundary. Subsequently, one would not anticipate that PD patients’ perceptions of their action boundaries would substantially differ from healthy older adult controls.

Another important factor to consider is that when patients are in an “off” phase, their ability to perform motor actions can be severely compromised to the extent that patients often report that they withdraw from society (Calne et al., 1996) and often simply do not perform motor actions. Subsequently, the patient may only obtain perceptual-motor experience regarding the maximal extent to which they can perform these actions whilst they are in an “on” phase, rather than obtaining variable perceptual motor experience in both “on” and “off” phases. Consequently, the patients’ perceptual motor experience regarding their ability to perform these actions will not be subject to random variability. Therefore, when asked to estimate the maximal extent to which they can perform these actions, the patient will calibrate to the consistent perceptual-motor experience obtained during “on” phases.

With regard to the underlying brain mechanisms, in PD, the degeneration of dopaminergic cells in the substantia nigra pars compacta initiates a cascade of functional changes affecting all basal ganglia structures (Blandini et al., 2000). Therefore, the findings obtained here may be taken to suggest that the basal ganglia do not affect the ability to judge one’s action capabilities and generate MI. However, it is possible that individuals with PD may use an alternative compensatory mechanism to ensure this ability remains intact. For example, it may be that individuals with PD rely more heavily on visual processing. Such that, rather than instinctively rapidly estimating their action capabilities, they may draw on conscious motor imagery processes, and take their time in making estimations as to whether the performance of an action would be successful or not. To the authors’ best knowledge, this is the first analysis of the influence of neurological conditions and altered neural processing on individual’s perceptions of their action capabilities. Therefore, to further inform our understanding of the underlying mechanism of anticipating one’s action capabilities, further work using this task with alternative patient groups (e.g., Huntington’s disease and focal brain injury patients) is required.

These findings have important implications for individuals suffering with mild-to-moderate PD. Despite the reduction in their ability to perform actions and variability in perceptual-motor experience that occurs in PD, individuals’ ability to accurately perceive their action boundaries for their upper limbs is preserved. Therefore, one can reasonably assume that they can use this knowledge to move safely within their environment. Physiotherapists and occupational therapists working with people with PD, may also draw upon this observation. It is important to highlight that individuals with PD may have developed a compensatory mechanism to preserve this function. Therefore, future research should investigate the method employed by people with PD when they perceive their action boundaries. Additionally, all tasks employed within this study focus solely on the perception of one’s action capabilities for upper body actions. As the execution of different motor actions is different mechanically and will have a differential demand upon the body (Jeannerod, 1996), it would be unreasonable to assume that the results obtained in this study can be generalized to the perception of action capabilities relating to both upper and lower body actions. Therefore, future research should analyze the perception of lower body action capabilities in PD. Finally, as all individuals with PD analyzed here display mild-to-moderate PD, it would be particularly interesting to analyze whether action boundary perception is less accurate in those with more severe motor symptoms.

In summary, these studies demonstrate that natural variability in one’s perceptual-motor feedback, as a consequence of PD, does not influence one’s subsequent perceptions of their action boundaries for reaching, grasping, and aperture passing. This implication is principally supported in the lack of significant difference (and support for the null using BF) between PD patients’ perceptions of their action capabilities and healthy older adult controls’ perceptions of their action capabilities. This finding may in part be due to the notion that typically PD patients spend a greater proportion of their waking hours in an “on” phase as opposed to an “off” phase. This result may also be explained by the notion that when PD patients are in an “off” phase, they characteristically do not perform actions and rather withdraw themselves from daily activities. Hence, they have little conflicting perceptual motor information specifying their action boundaries from when they are in their “on” phase.

These findings have important implications for people with PD. Specifically, as the results obtained indicate that individuals with PD’s ability to accurately perceive their action boundaries is preserved. One can reasonably assume that individuals with PD’s ability to use this information to ensure safe interaction with their environment remains intact. However, as all tasks employed here exclusively consider upper body actions these conclusions may be exclusive to the perception of upper body action capabilities.

### Supplementary Information

ESM 1 (SAV 34 kb)

## Data Availability

All data generated or analyzed during this study are included in this published article [and its supplementary information files].

## Appendix 1

### Analysis of covariance—The influence of anxiety on the perception of action capabilities

#### Task 1: Perception of reaching ability

Analyses revealed that there was no significant difference in the accuracy of the perceived action boundaries for reaching between the PD (*M*_*acc*_ = 1.050, *SE*_*acc*_ = .028) and healthy older adult groups (*M*_*acc*_ = 1.093, *SE*_*acc*_ = .025) after controlling for the effect of anxiety, although the evidence still only weakly supported the null, *F*(1, 37) = 1.278, *p* = .266, *B*_*H(0,0.09)*_ = 0.67, *RR*[0, 0.20]).

#### Task 2: Perception of grasping ability

Analysis revealed that there was no significant difference in the accuracy of the perceived action boundaries for grasping between the PD (*M*_*acc*_ = 1.017, *SE*_*acc*_ = .028) and healthy older adult groups (*M*_*acc*_ = 1.011, *SE*_*acc*_ = .026) after controlling for the effect of anxiety, although the Bayes factor now indicated that the evidence was now only weakly favoured the null, *F*(1, 37) = .030, *p* = .864, *B*_*H(0,0.08)*_ *=* 0.40, *RR*[0, 0.09]).

#### Task 3: Perception of aperture passing

Analysis revealed that there was no significant difference in the accuracy of the perceived action boundaries for aperture passing between the PD (*M*_*acc*_ = 1.043, *SE*_*acc*_ = .022) and healthy older adult groups (*M*_*acc*_ = 1.053, *SE*_*acc*_ = .021) after controlling for the effect of anxiety, although the Bayes factor still indicated that the data only weakly favoured the null hypothesis, *F*(1, 39) = .093, *p* = .762, *B*_*H(0,0.08)*_ *=* 0.39, *RR*[0, 0.09]).

## Appendix 2

### Analysis of covariance—The influence of the presence of rheumatic illnesses on the perception of action capabilities

#### Task 1: Perception of reaching ability

Analyses revealed that there was no significant difference in the accuracy of the perceived action boundaries for reaching between the PD (*M*_*acc*_ = 1.050, *SE*_*acc*_ = .028) and healthy older adult groups (*M*_*acc*_ = 1.093, *SE*_*acc*_ = .025) after controlling for the effect of the presence of rheumatic illness, *F*(1, 37) = 1.278, *p* = .266.

#### Task 2: Perception of grasping ability

Analysis revealed that there was no significant difference in the accuracy of the perceived action boundaries for grasping between the PD (*M*_*acc*_ = 1.017, *SE*_*acc*_ = .028) and healthy older adult groups (*M*_*acc*_ = 1.011, *SE*_*acc*_ = .026) after controlling for the effect of anxiety, although the Bayes factor now indicated that the evidence was now only weakly favoured the null, *F*(1, 37) = .030, *p* = .884.

#### Task 3: Perception of aperture passing

Analysis revealed that there was no significant difference in the accuracy of the perceived action boundaries for aperture passing between the PD (*M*_*acc*_ = 1.043, *SE*_*acc*_ = .022) and healthy older adult groups (*M*_*acc*_ = 1.053, *SE*_*acc*_ = .021) after controlling for the effect of anxiety, although the Bayes factor still indicated that the data only weakly favoured the null hypothesis, *F*(1, 39) = .093, *p* =.787.

## Appendix 3

**Table:**

	Actual reaching ability	Perceived reaching ability	Actual Grasping ability	Perceived grasping ability	Actual aperture passing ability	Perceived aperture passing ability
Years since diagnosis	−.288	−.370	−.274	−.173	.038	−.050
Years on medication	−.328	−.374	−.256	−.183	.059	−.042
Time since last dosage of medication (minutes)	−.134	.364	.063	.315	.127	−.051
LEDD	.308	−.461	−.266	−.244	−.005	−.189
UPDRS motor examination	.200	−.167	.087	.014	.213	−.157
UPDRS motor complications	.297	−.208	−.082	.107	−.031	.021
HADS–Anxiety Score	−.138	−.165	−.287	−.217	−1.44	.091
HADS– Depression Score	.097	−.001	−.041	−.063	−.061	.118
Rheumatic	−.196	−.004	−.361*	−.002	.025	.239

*Pearson correlations between saverage estimated/actual reaching ability ratio and Parkinson’s disease characteristics*

## Footnotes

1 The model of H1 was specified using differences in arm length between 10-year-olds and 18-year-olds reported by Živičnjak et al. (2003; *M*_*diff*_ = 16.70) as an upper limit of the extent arm reach may have differed between conditions.

2 Model of H1 specified using the results of Graydon et al. (2012, Experiment 1) who interpreted a difference in reaching accuracy ratios of 0.09 as evidence for the alternative hypothesis.

3 Model of H1 specified using the range of pinch grip aperture (13 cm to 21 cm, range: 8) reported by Holt et al. (2013) as a maximum upper limit of differences expected in reach aperture across conditions.

4 Model of H1 specified using the results of Graydon et al. (2012, Experiment 2).

5 Model of H1 specified using the room-to-move heuristic outlined by Dienes (2019).
